# Therapeutic Potential of Targeting ß-Arrestin

**DOI:** 10.3389/fphar.2019.00124

**Published:** 2019-03-06

**Authors:** Richard A. Bond, Emilio Y. Lucero Garcia-Rojas, Akhil Hegde, Julia K. L. Walker

**Affiliations:** ^1^ Department of Pharmacological and Pharmaceutical Sciences, College of Pharmacy, University of Houston, Houston, TX, United States; ^2^ School of Nursing, Duke University, Durham, NC, United States; ^3^ School of Nursing and Pulmonary Medicine, Duke University, Durham, NC, United States

**Keywords:** ß-arrestin, ß-arrestin signaling, G-protein-coupled receptors, biased signaling, 7TMR

## Abstract

ß-arrestins are multifunctional proteins that modulate heptahelical 7 transmembrane receptors, also known as G protein-coupled receptors (GPCRs), a superfamily of receptors that regulate most physiological processes. ß-arrestin modulation of GPCR function includes termination of G protein-dependent signaling, initiation of ß-arrestin-dependent signaling, receptor trafficking to degradative or recycling pathways, receptor transactivation, transcriptional regulation, and localization of second messenger regulators. The pleiotropic influence ß-arrestins exert on these receptors regulates a breadth of physiological functions, and additionally, ß-arrestins are involved in the pathophysiology of numerous and wide-ranging diseases, making them prime therapeutic targets. In this review, we briefly describe the mechanisms by which ß-arrestins regulate GPCR signaling, including the functional cellular mechanisms modulated by ß-arrestins and relate this to observed pathophysiological responses associated with ß-arrestins. We focus on the role for ß-arrestins in transducing cell signaling; a pathway that is complementary to the classical G protein-coupling pathway. The existence of these GPCR dual signaling pathways offers an immense therapeutic opportunity through selective targeting of one signaling pathway over the other. Finally, we will consider several mechanisms by which the potential of dual signaling pathway regulation can be harnessed and the implications for improved disease treatments.

## ß-Arrestin Structure and Function

The arrestin family of proteins includes four members and a variety of splice variants. There are two visual arrestins, found only in the retina (Smith et al., 2000), and two ubiquitously expressed arrestins named ß-arrestin1 (arrestin-2) (Lohse et al., 1990) and ß-arrestin2 (arrestin-3) (Attramadal et al., 1992). The beta prefix is because their first documented receptor substrate was the ß_2_-adrenergic receptor (ß_2_AR) and the “arrestin” term was because their major function is in terminating (or ‘arresting’) signaling *via* G proteins (Benovic et al., 1987). ß-arrestin proteins have functional and structural domains that allow them to bind to receptors as well as biochemical intermediates, events that are key to their function (Shenoy and Lefkowitz, 2011; Peterson and Luttrell, 2017). Although ß-arrestins interact with several different types of cell surface receptors (Shenoy and Lefkowitz, 2011), this review will focus on their role in modulating heptahelical receptors.

Heptahelical receptors, also known as seven-transmembrane receptors (7TMRs), are characterized by seven membrane-spanning domains and constitute the largest family of cell surface receptors known to date. The 7TMR superfamily is responsible for transducing a wide variety of extracellular signals into intracellular functions. More than three decades ago, it was shown that ligand activation of 7TMRs resulted in intracellular signaling through receptor coupling to heterotrimeric guanine nucleotide or G proteins (Rodbell et al., 1971; Northup et al., 1983). Thus, 7TMRs are more commonly known as G protein-coupled receptors (GPCRs). Therapeutically, these GPCRs are a very important class of receptor since they mediate almost all physiological processes, and their signaling is the target of roughly 40% of all prescribed drugs (Wise et al., 2002).

Numerous *in vitro* studies show that ß-arrestins terminate G protein-mediated cell signaling by translocating and binding to GRK-phosphorylated serine and threonine residues in the GPCR third intracellular loop or C terminal tail. Once bound, ß-arrestins sterically prevent further receptor-G-protein coupling (Benovic et al., 1987). This termination of G protein signaling is the canonical role for ß-arrestin. Later it was found that in addition to terminating G protein signaling, ß-arrestins also initiate ß-arrestin-dependent cell signaling by acting as scaffold proteins that couple the receptor to a growing list of signaling intermediates, many of which are kinases (Luttrell et al., 1999; Lefkowitz and Shenoy, 2005). This receptor-ß-arrestin-kinase complex is internalized *via* endocytic vesicles thereby becoming an intracellular “signalosome”. Recently, this concept has been broadened to show that endocytic vesicles lacking the receptor, but containing receptor-activated ß-arrestin, can also internalize and activate cell signaling (Eichel et al., 2018). In this newly discovered mechanism, the interaction of translocated ß-arrestin with the GPCR is temporary but sufficient to change the conformation of ß-arrestin thereby activating it. The activated ß-arrestin is able to bind to membrane phosphoinositides that link it to the cell membrane for endocytosis into signaling vesicles.

Internalization of signaling vesicles is mediated by interactions between the C terminal tail of GPCR-activated ß-arrestin and the cell membrane endocytic proteins, clathrin and AP-2 (adapter protein-2) (Goodman et al., 1996; Laporte et al., 1999). This adaptor function of ß-arrestin is crucially important to not only receptor internalization but also ß-arrestin-dependent signaling. ß-arrestins bind to E3 ubiquitin ligases and deubiquitinases that direct GPCR-ß-arrestin vesicles to degradative or recycling pathways within the cell, thus modulating receptor cell surface expression (Shenoy et al., 2001; Shenoy, 2007). This trafficking function of ß-arrestins is yet another way by which these multifunctional proteins modulate GPCR signaling and cellular responses. Furthermore, ß-arrestins are also able to dampen G protein-mediated second messenger generation (ie., cAMP) by binding to second-messenger degrading enzymes, such as phosphodiesterases (PDE), and translocating them to the ligand-activated receptor (Perry et al., 2002). Taken together, ß-arrestins are the predominant modulators of GPCR signaling. The activated conformation, and thus function, of ß-arrestin is influenced by the GPCR type to which it has translocated as well as the conformation of the activated receptor (reviewed in (Peterson and Luttrell, 2017)). The ß-arrestins are structurally flexible proteins which allow them to bind to a broad spectrum of partners and mediate a wide variety of functions (Scheerer and Sommer, 2017). Because biased ligands influence different conformational states of GPCRs, they indirectly influence ß-arrestin-dependent signaling, making them key therapeutic molecules. Additionally, biasing the structure/function of ß-arrestins to impact signal transduction has also been proposed as a novel therapeutic strategy (Chen et al., 2018). Our review does not focus on the finer points of structural data and complex equilibria of conformational states as these have recently been the topics of several excellent reviews (Peterson and Luttrell, 2017; Scheerer and Sommer, 2017; Chen et al., 2018).

With the development of mice deficient in either ß-arrestin1 or ß-arrestin2 (Conner et al., 1997; Bohn et al., 1999b), evidence supported the *in vitro* experimental conclusions that ß-arrestins play an important role in regulating normal physiological and pathophysiological responses. Despite the lethality resulting from elimination of both ß-arrestins, single ß-arrestin-knockout (ß-arrestin-KO) mice are quite normal and perturbations of homeostasis are often required to observe an effect of the absence of ß-arrestin expression.

The first published study examining normal physiologic responses in ß-arrestin2-KO mice involved exogenous opioid administration as the homeostatic perturbation (Bohn et al., 1999a). Bohn et al. showed the immediate anti-nociceptive effect of opioids was enhanced in ß-arrestin2-KO mice, and the negative side effects of opioids, such as respiratory depression and diminished gastrointestinal motility, were reduced. Although signaling pathways were not measured, the absence of depressed respiration and gut motility in ß-arrestin2-KO mice suggested that ß-arrestin-dependent signaling promoted those responses. Conversely, the enhanced analgesia pointed to a physiologically relevant role for ß-arrestin2-mediated desensitization of opioid receptor G protein-dependent signaling in the mechanism of pain relief.

The first study demonstrating a role for ß-arrestin in disease pathogenesis used a murine model of asthma (Walker et al., 2003). This study, and our subsequent work, showed ß-arrestin2-dependent phospho-p38 mitogen-activated protein kinase (Pp38) signaling to be crucial for T helper type 2 (Th2) cell chemotaxis (Lin et al., 2018). Furthermore, protection from developing the asthma phenotype in ß-arrestin2-KO mice is associated with significant inhibition of CD4+ Th2 cell chemotaxis to the lung and marked reductions in airway epithelial cell mucin secretion and airway inflammation (Walker et al., 2003).

## ß-Arrestin Modulation of Physiology and Pathophysiology

ß-arrestins trigger physiological responses through scaffolding of cell signaling proteins such as extracellular signal-regulated kinases 1 and 2 (ERK1/2), proto-oncogene tyrosine protein kinase Src (c-Src family tyrosine kinases), phosphoinositide 3-kinases, protein kinase B (AKT), c-Jun N-terminal kinases (JNK3) and elements of nuclear factor κb (NF κb) (Shenoy and Lefkowitz, 2011; Peterson and Luttrell, 2017).

Although convenient to characterize GPCR signaling as two distinct signaling pathways, one that is G-protein dependent/ß-arrestin-independent and the other that is ß-arrestin-dependent/G protein-independent, the reality is more complex. We recently showed that P-p38 signaling, and associated chemotaxis, of T helper type 2 cells is partially dependent on ß-arrestin2 and that this ß-arrestin2-dependent signaling is downstream of Gαi (Lin et al., 2018). In that paper, we described the dual chemotaxis signaling pathways as ß-arrestin dependent and ß-arrestin independent; of course, both pathways were G protein-dependent. Work from Grundmann et al. (2018) has provided further evidence of G protein-dependency for ß-arrestin-mediated signaling. They used groundbreaking CRISPR/Cas9 technology to produce HEK293 cells that either do not express arrestins or lack all G proteins except Gαi, which was pharmacologically inhibited using pertussis toxin (PTX). Using these cell lines, dubbed “zero arrestin” and “zero functional G,” respectively, they showed that ß-arrestin-dependent signaling is downstream of G proteins for several GPCRs including some canonical receptors where this was not previously believed to be the case. Although the receptor and cell types were limited in their study, the results have important implications for drug discovery and suggest G proteins as the “genuine drivers of GPCR-mediated signal transduction.” However, whether or not ß-arrestin is a signaling molecule or a scaffolding protein, or is dependent or independent of G protein signaling, is immaterial to its extremely important regulation of cellular function (Gutkind and Kostenis, 2018). Thus, the lexicon used to describe GPCR signaling could benefit from expansion and clarification where descriptors of both the G protein and ß-arrestin involvement are listed (G protein-dependent/independent and ß-arrestin-dependent/independent).

As predicted by experimental findings, the ß-arrestin-dependent signaling pathway, like its G protein (ß-arrestin-independent) counterpart, regulates a wide variety of important cellular responses including cell development, growth and survival, immune cell function, protein translation, and neuronal signaling (Gu et al., 2015). The ability of ß-arrestins to desensitize G protein-dependent and mediate ß-arrestin-dependent GPCR signaling pathways uniquely positions these proteins to exert a major influence on physiology and pathophysiology.

The discovery that a single GPCR subtype can couple to different transduction proteins and produce multiple cellular responses has led to development of ligands that can preferentially “bias” the receptor toward one pathway. Some of these “biased ligands” preferentially target the ß-arrestin pathways (by either activation or inhibition) and have shown promise in drug development. These advances will be discussed in the section titled *ß-arrestin versus G-protein signaling in disease*.

## ß-Arrestin Expression in Disease

Consistent with the broad range of physiological processes modulated by ß-arrestins, the upregulation of ß-arrestin expression is associated with many diseases. Whether or not changes in expression are adaptive or maladaptive remain to be determined. For example, in a mouse model of cardiac dysfunction, enhanced cardiac ß-arrestin2 expression mitigated adverse cardiac remodeling (Grisanti et al., 2018); whereas in murine asthma, T cell and lung structural cell overexpression of ß-arrestin2 is maladaptive (Walker et al., 2003; Chen et al., 2015; Sharma and Parameswaran, 2015). Elevated ß-arrestin expression and concomitant anti-apoptotic effect is associated with fibrotic diseases (reviewed in (Gu et al., 2015) and in multiple sclerosis (MS) where CD4+ T cells from patients have a higher expression of ß-arrestin1 (Shi et al., 2007). These results implicate increased ß-arrestin1 as mediating the survival of CD4+ T and promoting disease pathogenesis (Shi et al., 2007). Another study reported similar upregulation of ß-arrestin1 expression in the brains of MS patients and in an animal model of experimental autoimmune encephalomyelitis (EAE), a commonly used model of human inflammatory demyelinating disease (Tsutsui et al., 2008). Other diseases that implicate ß-arrestin in pathophysiology include Alzheimer’s disease (AD), cystic fibrosis (CF) and meningitis. In AD, brain protein and mRNA levels of ß-arrestin1 and ß-arrestin2 are elevated (Liu et al., 2011; Jiang et al., 2013). In CF, patient nasal epithelial cells, as well as CF model cells, overexpress ß-arrestin2 (Manson et al., 2008). With respect to immunity, expression of ß-arrestin2 was shown to be elevated in PBMCs of patients with cryptococcal meningitis (Bochaton-Piallat et al., 2016). Up and down-regulation of ß-arrestin proteins is clearly associated with pathology. Interestingly, disease-associated mutations in either ß-arrestin subtype have, so far, not been found.

## Effects of ß-Arrestin on Disease

Inappropriate modulation of cell survival can lead to cancer and fibrotic diseases, while abnormalities in immune cell function have implications for autoimmunity, infection, and the inflammatory component of many diseases. For an in depth review of the role for ß-arrestins in disease, please refer to the work by Sharma and Parameswaran (2015). Below we present examples of how ß-arrestin modulation of cellular events can become pathophysiological.

Once adhered to human brain endothelial cells, the meningococcus bacterium promotes endothelial cell ß_2_AR-ß-arrestin2 signaling to cSrc-mediated cytoskeletal reorganization (Coureuil et al., 2010). This delocalizes endothelial junction proteins resulting in destruction of the blood brain barrier tight junctions and enhanced brain infection. Cytoskeletal changes also promote bacterial adhesion to endothelial cells (Coureuil et al., 2010). ß-arrestin2 also hinders bacterial killing through reducing peripheral blood mononuclear cell cytotoxic activity, decreasing serum levels of interferon-gamma (IFN-γ), an anti-bacterial cytokine, and increasing the serum level of IL-10, an anti-inflammatory cytokine (Bochaton-Piallat et al., 2016). Similarly, ß-arrestin2 inhibits the antiviral response by reducing the killing effectiveness of natural killer (NK) cells (Yu et al., 2008). ß-arrestin2 transgenic mice were shown to have higher organ viral loads following murine cytomegalovirus; additionally, NK cells from these mice displayed reduced cytotoxicity. Conversely, ß-arrestin2 knockout mice and their NK cells displayed lower tissue viral titers and enhanced cytotoxicity, respectively.

Studies have also shown that ß-arrestins are involved in the initiation, development, and metastasis of many types of cancer (Song et al., 2018). In a murine model of chronic myelogenous leukemia, in which mice were transplanted with diseased hematopoietic stem cells, mice devoid of ßarrestin-2 did not succumb to the disease following transplantation of the diseased cells. In contrast, control mice died within 2 months of the transplantation of diseased cells. The study showed ß-arrestin2 promotes signaling *via* the wnt/beta-catenin pathway to promote cancer stem cell maintenance (Fereshteh et al., 2012). Similarly, in a murine model of myelofibrosis, a myeloproliferative neoplasm, mice transplanted with donor ß-arrestin2-KO hematopoietic stem cells infected with a myelofibrosis retrovirus did not develop the disease, whereas controls uniformly succumbed to disease. Abolition of ß-arrestin2-mediated promotion of anti-apoptosis prevented ß-arrestin2-KO cells from repopulating long-term and decreased self-renewal of infected ß-arrestin2-KO cells (Rein et al., 2017).

The role of ß-arrestins in ovarian, prostate, brain, gastric, lung, and breast cancers has been well established (Sobolesky and Moussa, 2013). One study showed ß-arrestin1 could be used as a plasma biomarker to differentiate certain types of lung cancers (El-Khoury et al., 2018). Ovarian cancer metastasis has also been reported to be mediated by ß-arrestin1 (Purayil and Daaka, 2018). Studies also suggest ß-arrestin2 as a prognosis marker for colorectal cancer (Ren et al., 2018) and a promoter of lymph node metastasis in non–small cell lung cancer (Cong et al., 2017). Both ß-arrestin1 and 2 have been reported as promoters of prostate cancer albeit through different mechanisms (Kong et al., 2018). Numerous GPCRs and their downstream effectors are envisaged to provide many targets and novel strategies in hepatocellular carcinoma prevention and treatment (Peng et al., 2018). Interestingly, the ß-arrestin biased ß-blocker carvedilol, a ligand that activates ß_2_AR-ß-arrestin2 signaling while inactivating canonical ß_2_AR-Gs signaling, has been found to be beneficial in cancer prevention by virtue of blocking a key step in carcinogenesis, i.e., ERK translocation into the nucleus (Wisler et al., 2007; Cleveland et al., 2018).

Fibrosis is defined as the accumulation of excess extracellular matrix (ECM) components which are mainly derived from fibroblasts and myofibroblasts (Cox and Erler, 2011; Bochaton-Piallat et al., 2016). If highly progressive, the fibrotic process leads to organ malfunction and death (Gu et al., 2015). Renal fibrosis can result in many kidney diseases (diabetic nephropathy, uronephrosis and polycystic kidney) and often leads to chronic kidney disease. ß-arrestin-deficient murine models under unilateral ureteral obstruction show attenuated renal interstitial fibrosis. Moreover, mice and human kidney tissue samples morphologically consistent with nephropathy show increased expression of ß-arrestin1 (Xu et al., 2018). However, it is unknown what receptor is responsible for such a response. Data have implicated AT_1_ receptor-mediated ß-arrestin signaling as responsible for the observed increased extracellular matrix synthesis resulting in renal fibrosis (Wang et al., 2017b). Mice with unilateral ureteral obstruction had increased collagen I and fibronectin that correlated with the tubulointerstitial fibrosis and ß-arrestin upregulation. When ß-arrestin signaling was stimulated in a rat renal fibroblast cell line (NRK-49F cells) using [Sar(1), Ile(4), Ile(8)] AngII (SII), an AT_1_ receptor ß-arrestin biased ligand, increased collagen I and fibronectin expression was observed, while silencing ß-arrestin expression had the opposite effect (Wang et al., 2017b). Expression of ß-arrestin2 is upregulated in liver biopsies from patients with hepatitis B and C, suggesting ß-arrestin2 promotes liver fibrosis (Gu et al., 2015). Taken together, the above data suggest ß-arrestin signaling promotes fibrosis in some tissues such as kidney, liver, heart, and lung (Lovgren et al., 2011; Gu et al., 2015). These results support the development of G-protein biased ligands that may shut down the pro-fibrotic actions of ß-arrestin in disease.

The range of diseases in which ß-arrestins play a role is very broad. For example, ßarrestin plays a role in brain function. A positive correlation between brain ß-arrestin (ß-arrestin1 and 2) levels (both protein and mRNA) and Alzheimer’s disease diagnosis, severity, and amyloid burden has been demonstrated by several investigators (Liu et al., 2011; Thathiah et al., 2012; Liu et al., 2013). Also, G protein-independent signaling downstream of brain β2AR, delta opioid receptor, and orphan G protein-coupled receptor 3 promote cleavage of amyloid precursor protein (APP) by γ-secretase (Jiang et al., 2013) and production of amyloid-β peptide, a defining pathological feature of AD. In a murine model of AD, neurons lacking ß-arrestin2 demonstrate reduced amyloid-ß peptide secretion in culture and detection in the hippocampus and cortex (Thathiah et al., 2012). In brief, ß-arrestin 2 promotes the pathogenesis of AD through GPCR-initiated regulation of γ-secretase activity, which results in elevated levels of amyloid-ß peptide (Jiang et al., 2013). In other cell types, ß-arrestin can have a positive impact on brain function. For example, ß-arrestin2 promotes cofilin translocation to dendritic spines in response to N-methyl-D-aspartic acid (NMDA) receptor activation, and cofilin promotes dendritic spine remodeling which is needed for normal learning and memory (Pontrello et al., 2012).

There are also examples where the canonical role for ß-arrestins is involved in neuropathophysiology. In Parkinson’s disease (PD), a neurodegenerative illness where loss of dopaminergic neurons from the nigrostriatal system affects locomotion, chronic treatment with L-DOPA, a dopamine precursor, induces dyskinesias by D_1_ receptor overactivation (Urs et al., 2015). These abnormal involuntary movements are mediated by Gs signaling as noted by rodent and nonhuman primate models of PD. In ß-arrestin2-KO mice treated with L-DOPA, such movements increased after L-DOPA treatment compared to controls and were prevented after ß-arrestin2 overexpression in both mice and monkeys (Urs et al., 2015). Using PD animal models (rats and macaques), others have demonstrated that using Gs-biased ligands for D_1_ receptors that decrease βarrestin-2 recruitment and associated desensitization of G protein signaling results in sustained locomotive activity (Gray et al., 2018), supporting a canonical role for ß-arrestin in regulating PD dyskinesias.

## ß-Arrestin Versus G-Protein Signaling in Disease

As discussed above, data from the last 2 decades have shown that a single GPCR subtype can couple to different transduction proteins and produce multiple cellular responses. These observations have resulted in a new era of pharmacology where, relative to the endogenous hormone or neurotransmitter, ligands can selectively or at least preferentially activate one of the diverse responses produced by a single GPCR subtype. This phenomenon has been termed “ligand-directed trafficking of receptor stimulus” or “biased signaling.” In many cases, the therapeutic effect of a ligand is mediated by one pathway and the adverse effects by another pathway. Thus, rational development of biased ligands has become an active part of modern drug discovery.

To date, the diseases with the most clinical data in support of the development of biased drugs are: the use of ß-adrenergic receptors (ßAR) and angiotensin AT_1_ receptor ligands in congestive heart failure (CHF) (Barrese and Taglialatela, 2013; Lymperopoulos and Aukszi, 2017); μ-opioid receptor (μOR) ligands in the management of pain; and to a lesser extent, possibly ß_2_AR ligands in asthma (Dickey et al., 2010; Forkuo et al., 2016; Joshi et al., 2017; Nguyen et al., 2017). There is also strong preclinical evidence supporting the use of biased ligands for other diseases, such as D_1_ and D_2_ receptor ligands for Parkinson’s disease and schizophrenia, respectively (Park et al., 2016; Gray et al., 2018), sphingosine 1P (S1P) receptor ligands for multiple sclerosis (Dhar et al., 2016), and adenosine A_1_ receptor ligands for ischemic heart disease (Baltos et al., 2016), among others. It is important to emphasize that neither the canonical signaling pathway associated with the G-protein nor signaling *via* the ß-arrestin-dependent pathway (or any pathway for any receptor) can be termed as universally beneficial or detrimental. The (patho)-physiological effects of any pathway will always be disease-specific, time-dependent, and indeed often cell-specific.

For example, in CHF and asthma, the pathways mediating the beneficial versus adverse effects are currently believed to be the opposite for each disease. In CHF, there are data to support ß-arrestin signaling as anti-apoptotic and cardioprotective (Rojanathammanee et al., 2009; Carr et al., 2016b). This is not only true for ß-arrestin activation by the FDA-approved “ß-blocker,” carvedilol, in CHF, but also ß-arrestin activation in other GPCRs such as the angiotensin AT_1_ receptor (Kim et al., 2012; Monasky et al., 2013). In CHF, the angiotensin AT_1_ receptor can activate both its canonical G-protein pathway (in this case, Gq) and the ß-arrestin-dependent pathway (Noor et al., 2011; Monasky et al., 2013; Teixeira et al., 2017). Considerable data implicated a clear division between the detrimental effects of AT_1_ receptor signaling via Gq and the protective effect of signaling via ß-arrestin and led to the development of the biased antagonist, TRV-120027 that preferentially inhibits Gq signaling (Boerrigter et al., 2011; Boerrigter et al., 2012). Thus, the ideal ligand profiles for both the ß_2_AR and AT_1_ receptor, when used in CHF, appear to be ligands that antagonize the G-protein pathway (Gs and Gq respectively), and either stimulate, or at least not antagonize the receptor conformation that promotes ß-arrestin-dependent signaling. Perhaps the most compelling evidence for the advantage of this ligand profile as ideal is the observed therapeutic advantage of carvedilol in CHF (Wisler et al., 2007). In this regard, it is important to point out a study using a mutated ß_2_AR (ß_2_AR^TYY^) where the mutation renders the ß_2_AR unable to bind G proteins; carvedilol was the only beta-blocker that retained the ability to activate ERK1/2 (Shenoy et al., 2006). However, more recent findings show that initiation of ERK1/2 activation by ß_2_AR involves a signaling route that is independent of ß-arrestins (O’Hayre et al., 2017). Examination of the role for ß-arrestins in ß_1_AR signaling to ERK1/2 shows that all ERK1/2 signaling downstream of the ß_1_AR requires Gαi protein activation (Wang et al., 2017a), including that induced by carvedilol, but that loss of ß-arrestin2 results in reduced ERK1/2 signaling (Eichel et al., 2016). Future studies are required before we fully understand how carvedilol’s superior clinical efficacy may be related to its unique signaling profile and the role for ß-arrestins. For now, O’Hayre et al. posit that re-interpretation of original findings with respect to the impact of the scaffolding function of ß-arrestins, which may control the localized activation of ERK, versus the ß-arrestin-promoting activation of ERK1/2 may explain some discrepancies (O’Hayre et al., 2017).

In the management of pain, μ opioid receptors (μOR) mediate pain relief through Gi/o activation, while ß-arrestin-dependent signaling induces respiratory depression and constipation (Bohn et al., 1999b; Raehal et al., 2005; DeWire et al., 2013; Altarifi et al., 2017). For example, compared to their wild type controls, ß-arrestin2-KO mice treated with morphine, a μOR agonist, exhibited increased antinociception using a tail-flick and hotplate models of pain (Bohn et al., 1999b). In addition, the ß-arrestin2-KO mice exhibited less of a reduction in gastrointestinal transit, and no respiratory suppression was observed compared to wild type mice (Raehal et al., 2005). These and other findings led to the development of TRV130, also known as oliceridine, a Gi/o-biased ligand (DeWire et al., 2013; Altarifi et al., 2017) for the μOR. Clinical trials using TRV130 for phase II (Viscusi et al., 2016; Singla et al., 2017) and III studies (APOLLO-1 and -2) have been completed, and while the results are not yet published, TRV-130 displayed greater analgesia and less gastrointestinal and respiratory side effects compared to morphine (Fossler et al., 2018). Currently, an open-label safety study is underway (ATHENA trial).

Thus, clinical development of biased ligands is now an active area of research by several pharmaceutical companies and academic laboratories. However, to date, no new chemical entities have made it to FDA-approval, and some have failed in Phase II trials (Pang et al., 2017). While this may cause diminished enthusiasm that pursuing the development of biased ligands may only work theoretically, it is important to emphasize that biased ligands such as carvedilol have already shown superior clinical efficacy (Wisler et al., 2007). There are also data showing, famotidine, a histamine H_2_ receptor antagonist used to reduce gastric acid secretion in acid-peptide disorders, may have greater therapeutic efficacy compared to other H_2_ receptor antagonists (Campoli-Richards and Clissold, 1986). A suggested explanation is that famotidine, besides working as a G protein signaling antagonist by decreasing cAMP, also stabilizes the H_2_ receptor conformation that induces desensitization, likely through ß-arrestin (Alonso et al., 2015).

The second generation antipsychotic cariprazine, a dopamine D_2_ and D_3_ receptor partial agonist in most systems, was recently approved for the treatment of schizophrenia and exhibited increased safety and tolerability profiles compared to first generation antipsychotics (Durgam et al., 2015). Although not a selective drug, biased signaling towards Gi has been suggested to confer cariprazine with less side effects (i.e. cognitive impairment, hyperprolactinemia, weight gain) (Solmi et al., 2017). Conversely, preclinical data also suggest that ß-arrestin biased ligands for the D_2_ receptor can also contribute to schizophrenia treatment by resetting the balance of the excitation inhibition in the prefrontal cortex (Urs et al., 2016). Therefore, both signaling pathways are highly important in the therapeutics for this pathology and reinforce the idea that both targets are equally valuable in drug discovery.

As noted above, some recent attempts to rationally develop biased ligands have failed at various stages of development. However, this should not be interpreted as a failure that biased ligands can be developed. Indeed, one of the major societal and scientific benefits of the pharmaceutical industry’s development of dozens of generic drugs for major diseases and symptoms, is that biased ligands have already been developed. For example, the scientific community has already thoroughly screened, using *in vitro* assays, dozens of ß_2_AR and μOR ligands already in clinical use for their activity at several pathways (Wisler et al., 2007; Molinari et al., 2010; Stallaert et al., 2012; Vezzi et al., 2013; van der Westhuizen et al., 2014); and as described above, other drugs are now retroactively being implicated as being biased ligands as a means of explaining their different therapeutic outcome from other members of the same class of drugs (Wisler et al., 2007; Shonberg et al., 2013; Alonso et al., 2015).

To further develop the hypothesis that biased ligands can be rationally designed, it may be useful to view the strategy as not dissimilar from one previously used to produce dozens of therapeutically improved ligands. The last half of the 20^th^ century saw a proliferation of the discovery of receptor subtypes. In many ways, biased ligand synthesis can be viewed as analogous to the development of more receptor subtype selective ligands. The different conformational states that are thermodynamically required to activate different pathways can be viewed in the same way as the different receptor conformations associated with different receptor subtypes.

Alternative or complementary ways to bias GPCR signaling include allosteric modulation of GPCR as well as the use of pepducins and/or nanobodies. Nanobodies, camelid antibody fragments, have been developed that preferentially bind to and stabilize the human ß_2_AR in various conformations (Rasmussen et al., 2011; Staus et al., 2016). Rasmussen et al. produced nanobody 80 (Nb80) that, when bound to receptor, stabilizes the conformation of the receptor producing Gs signaling. Staus *et al* developed four families of nanobodies that stabilized active or inactive ß_2_AR conformations and found biased inhibition of either G protein activation or ß-arrestin recruitment (Staus et al., 2014). They identified Nb60, a negative allosteric nanobody, to modulate and stabilize inactive β2AR state (Staus et al., 2016). As nanobodies can modulate biased ß_2_AR signaling, they can be potential therapeutic agents regulating various pathological processes involving ß_2_AR. Interestingly, Martin et al. synthesized a set of peptidomimetics which are structurally similar to the complementarity-determining region 3 (CDR3) of the nanobody Nb80, and inhibit ß_2_AR-G protein coupling (Martin et al., 2017).

Another approach to biasing receptor signaling involves pepducins, first developed by Covic et al. (2002a,b). Pepducins are the lipid-peptide conjugates with sequences derived from the intracellular loops of the targeted GPCR (Carr and Benovic, 2016). By penetrating cells, pepducins can access receptor conformations not accessible to extracellular ligands that must rely on extracellular receptor binding (Carr et al., 2016b). Pepducins regulate the activity of GPCRs by allosteric modulation (Quoyer et al., 2013). Pepducins for several GPCRs have been reported in the last decade (Covic et al., 2002a; Licht et al., 2003; Remsberg et al., 2007; Tchernychev et al., 2010; Carr et al., 2014, 2016b). For example, intracellular loop1–9, a ß-arrestin–biased pepducin for the β_2_AR, has been reported to be completely ß-arrestin–biased in primary adult murine cardiomyocytes, possibly enhancing cardioprotective effects for CHF therapy (Carr et al., 2016b). Intracellular loops 3–9, a pepducin modulator of G_s_-biased ß_2_AR signaling, has been shown to be a potential asthma therapy candidate as ß-arrestins are believed to be responsible for the symptoms associated with asthma (Walker et al., 2003; Dickey et al., 2010; Thanawala et al., 2013; Carr et al., 2014; Lin et al., 2018). Another G_i_-biased pepducin, ATI-2341, has been developed for the chemokine receptor CXCR4 (Quoyer et al., 2013). It is noteworthy that successful use of pepducins *in vivo* is currently constrained because pepducins lack a targeting mechanism in multilayered tissues and thus are limited to cells in close proximity to the circulating pepducins (Carr et al., 2016a). However, pepducins can potentially share sequences in the intracellular loops of closely-related GPCRs and may have enhanced therapeutic effects mediated by various GPCRs (“polypharmacology”) (Carr and Benovic, 2016).

## Concluding Remarks

Given the functional versatility of ß-arrestins, they are aptly suited to effectively and broadly regulate cell signaling and resultant physiological and pathophysiological processes. The therapeutic potential of targeting ß-arrestins is enormous, since disease-specific treatments could increase the safety and efficacy of GPCR-targeted therapeutics. On the other hand, given that a single ß-arrestin subtype can modulate dozens of GPCRs, this may pose problems in drug discovery. The search for ß-arrestin modulators will not be easy given the complexity of GPCR signaling pathways and the pleiotropy of ß-arrestin functions making precise targeting of paramount importance. Despite these challenges, there are several ligands preferentially targeting ß-arrestin signaling now in clinical trials and more in development. Thus, we will hopefully soon have answers as to the impact of these ligands in future therapies.

## Author Contributions

JW and RB contributed to the conceptualization of the subject matter. JW, RB, ELG-R, and AH reviewed the literature and wrote sections of the manuscript.

### Conflict of Interest Statement

The authors declare that the research was conducted in the absence of any commercial or financial relationships that could be construed as a potential conflict of interest.
